# Frieden für wen? Sicherheitspolitik im Kontext der Gewalt gegen Menschenrechtsaktivist*innen in Kolumbien

**DOI:** 10.1007/s42597-021-00058-0

**Published:** 2021-07-14

**Authors:** Rosario Figari Layús

**Affiliations:** Justus Liebig Universität Giessen, Licher Str. 76, 35394 Gießen, Deutschland

**Keywords:** Sicherheit, Menschenrechtsverteidiger*innen, Kolumbien, Menschenrechte, Politische Gewalt, Security, Human rights defenders, Colombia, Human rights, Political violence

## Abstract

In den letzten Jahren, insbesondere seit der Unterzeichnung des Friedensabkommens zwischen der kolumbianischen Regierung und der FARC-Guerilla Ende 2016, sind Menschenrechts- und Umweltaktivist*innen in vielen Regionen Kolumbiens mit zunehmenden Angriffen und Einschüchterungen konfrontiert. In diesem Zusammenhang hat die kolumbianische Regierung eine Reihe von Maßnahmen implementiert, um dieser zunehmenden Gewalt zu begegnen. Dieser Beitrag analysiert, wie die dieser staatlichen Initiative zugrunde liegenden Konzepte von „Schutz“ und „Sicherheit“ sowie ihre Umsetzungsdynamiken schwierige Folgen für einen effektiven Schutz von gefährdeten Akteur*innen der Zivilgesellschaft in Kolumbien gebracht haben. Dafür werden die Rolle, die Auswirkungen und die Herausforderungen solcher Programme berücksichtigt, die oft Aktivist*innen nicht langfristig schützen können, sondern sogar viele der gefährdeten Akteur*innen ausschließen und oft sogar in größere Gefahr bringen. Welche staatlichen Initiativen werden in Kolumbien implementiert, um der zunehmenden Gewalt zu begegnen und die Zivilgesellschaft zu schützen? Welche Auswirkungen haben diese staatlichen Maßnahmen in Kolumbien bisher gezeigt und welche Herausforderungen sind immer noch zu identifizieren?

## Abstract (en)

In recent years, especially since the signing of the peace agreement between the Colombian government and the FARC guerrillas in 2016, social leaders, human rights and environmental activists and other critical groups have been facing increasing attacks and hostilities by state and non-state actors in many regions of Colombia. In this context, the Colombian government has implemented a series of security measures and programs to address this increasing violence and provide protection to social leaders at risk. Thus, this paper analyzes the conceptual framework of “protection” and “security” underlying these governmental initiatives as well as the implementation procedures, that have proven to have serious difficulties and failures in providing effective protection to threatened civil society actors. As this article shows, the Colombian security paradigm and protection policies for human rights activists and social leaders do not seem to have an effective long-term, comprehensive and participative approach, increasing even in some cases the risk of those who should protect. In order to discuss these points, this paper will address the following questions: What are the main characteristics and dilemmas of the Colombian state protection policy? How do these protection policies impact on the security of social leaders and civil society actors? What are the main challenges to effectively reduce violence against social leaders in Colombia?

## Einleitung

Seit Jahrzehnten sind Menschenrechts- und Umweltaktivist*innen in vielen Regionen Kolumbiens Angriffen, Einschüchterungen und Gewalt bis hin zu Mordattacken ausgesetzt. Dies hat seit der Unterzeichnung des Friedensabkommens zwischen der kolumbianischen Regierung und der FARC(„Fuerzas Armadas Revolucionarias de Colombia-Ejército del Pueblo“)-Guerilla 2016 weiter zugenommen. Zivilgesellschaftliche Organisationen werden täglichen mit Bedrohungen und Anfeindungen konfrontiert, hinter denen größtenteils staatliche beziehungsweise private Akteure stehen, die oft eng mit kriminellen Strukturen zusammenarbeiten. Daraus ergibt sich eine der zentralen Fragen dieses Artikels: Frieden für wen? Offensichtlich scheint der Frieden die Menschenrechtsaktivist*innen noch nicht erreicht zu haben. In diesem Zusammenhang hat der kolumbianische Staat unter den unterschiedlichen Regierungen Sicherheitspolitiken implementiert, die aus mehreren – oft widersprüchlichen – Schutzmaßnahmen und Programmen bestehen, um der zunehmenden Gewalt gegen zivilgesellschaftliche Akteure zu begegnen. In diesem Artikel, wie auch in Kolumbien, werden die Begriffe Sicherheitspolitik und Schutzpolitik als Synonym verwendet. Die Priorität der Schutzmaßnahmen liegt darauf, das Leben und die körperliche Unversehrtheit der Menschenrechtsverteidiger*innen zu schützen und ihnen damit zu ermöglichen, ihre Arbeit frei und ohne Gefahr fortzusetzen. Betrachtet man jedoch die aktuelle Anzahl von Angriffen, Drohungen und Morden an Aktivist*innen, stellt sich die Frage nach ihrer realen Wirksamkeit, Effektivität und Reichweite.

Auf internationaler Ebene wurden Erklärungen und Richtlinien sowohl von den Vereinten Nationen als auch auf regionaler Ebene, v. a. im interamerikanischen Menschenrechtssystem, zum Schutz von Aktivist*innen ausgearbeitet. Besonders relevant ist die „Erklärung der Vereinten Nationen über das Recht und die Verantwortung von Einzelpersonen, Gruppen und Organen der Gesellschaft zur Förderung und zum Schutz von allgemein anerkannten Menschenrechten und Grundfreiheiten“ (1998). Die Erklärung konstatiert, dass der Staat für die Sicherheit dieser Menschen verantwortlich ist (VN-Deklaration zum Schutz von Menschenrechtsverteidigern 1998). Allerdings entscheiden die Staaten, wie sie diese Aufgabe erfüllen wollen.

Dieser Beitrag analysiert mit Fokus auf die Zeit nach dem Friedensabkommen (ab 2016) die Hauptmerkmale und Herausforderungen der umstrittenen kolumbianischen Sicherheitspolitik zum Schutz von gefährdeten Gruppen. Der Schwerpunkt liegt auf den in Kolumbien sogenannten *líderes sociales*, ein Begriff, der sich auf Vertreter*innen sozialer und politischer Gruppen und kritischer sozialer Bewegungen, Umwelt- und Menschenrechtsverteidiger*innen sowie Journalist*innen bezieht. Hierfür werden zwei Instrumente der kolumbianischen Schutzpolitik in den Fokus gerückt: a) der Plan zur angemessenen Aktion (*Plan de Acción Oportuno* – im Folgenden PAO) und b) die Nationale Schutzeinheit (*Unidad Nacional de Protección* – im Folgenden UNP). Der PAO ist die Schutzstrategie auf Makro- bzw. nationaler Ebene und wurde von der aktuellen Regierung unter Iván Duque im November 2018 eingeführt. Im Ergebnis wurden die Regionen mit den meisten Angriffen auf soziale Aktivist*innen militarisiert und die kolumbianischen Streitkräfte gestärkt. Als Maßnahme auf Mikro- bzw. individueller Ebene wird die UNP analysiert, ein staatliches Programm des Innenministeriums, das bedrohten Menschenrechtsverteidiger*innen und anderen gefährdeten Gruppen wie etwa Journalist*innen, Gewerkschafter*innen, Justizakteur*innen und Politiker*innen Schutzmaßnahmen anbietet. Dieses Programm wurde bereits 1998 verabschiedet und 2011 von der Regierung Juan Manuel Santos (2010–2018) umstrukturiert. Hierbei stellen sich folgende Fragen: Welche Rolle und Auswirkungen haben staatliche Schutzmaßnahmen in Kolumbien bei der Bekämpfung und Beendigung von Gewalt? Welche Eigenschaften und Herausforderungen weist diese Schutzpolitik auf? Welche Konsequenzen haben die Schutzmaßnahmen tatsächlich für ihre Zielgruppe?

Eines der Hauptargumente dieses Textes ist, dass die mangelnde Wirksamkeit und die Herausforderungen der Schutzpolitik auf ein historisches und konfliktives Verhältnis zwischen dem kolumbianischen Staat und kritischen sozialen Akteuren der Zivilgesellschaft zurückzuführen sind. Diese historisch verankerte antagonistische Beziehung zwischen sozialen Aktivist*innen und dem Staat hat sich in zahlreichen Maßnahmen und Kampagnen zur Diskreditierung und Stigmatisierung, in mangelndem Dialog und sogar Kriminalisierung der Menschenrechtsverteidiger*innen niedergeschlagen. Während die Regierung Santos eine offenere und aufgeschlossenere Haltung zum Dialog zeigte, wurden die Spannungen durch die Regierung Iván Duque noch einmal verstärkt. In diesem Zusammenhang stellt sich die Frage, wie die von Gewalt betroffenen Aktivist*innen die Schutzumaßnahmen wahrnehmen und wie sich aus deren Perspektive das Scheitern dieser erklären lässt?

Die Analyse der Effektivität und Komplexität der Schutzpolitik fokussiert die Perspektive ihrer Hauptnutzer*innen und ihrer Zielgruppe: Vertreter*innen sozialer Organisationen, Aktivist*innen und Journalist*innen. Die Perspektive der Betroffenen spielt eine grundlegende Rolle, um die Funktionsweise und die Herausforderungen der Schutzpolitik zu verstehen. Der Beitrag basiert auf Sekundärliteratur, journalistischen Quellen, offiziellen Mitteilungen und Dokumentationen von Nichtregierungsorganisationen und der Auswertung von 14 semi-strukturierten Interviews mit Vertreter*innen von zivilgesellschaftlichen Organisationen in Bogotá und Medellín aus dem Jahr 2019. Der Beitrag analysiert vor allem die subjektiven Wahrnehmungen der befragten Betroffenen von Gewalt und Bedrohungen. Damit gewinnt die Analyse in der empirischen Herausarbeitung der induktiv erhobenenen Faktoren ihren erklärenden Mehrwert. Die Gruppe der Befragten wurde mithilfe des Schneeball-Samplings zu einer Stichprobe zusammengefasst (Biernacki und Waldorf 1981; Atkinson und Flint 2003). Ein Schlüsselkriterium für die Auswahl der Befragten war, dass sie ein möglichst breites Spektrum von verschiedenen Arten von Aktivismus wiederspiegeln (bspw. Mitglieder von etablierten Menschenrechtsorganisationen, Journalist*innen, Gewerkschafter*innen, Aktivist*innen von Basisorganisationen). Um die Vertraulichkeit und die Sicherheit der befragten Personen zu gewährleisten, werden ihre Namen und die ihrer jeweiligen Organisationen anonymisiert.

Der Aufsatz ist in drei Abschnitte gegliedert. Zunächst wird der Kontext analysiert, in dem Vertreter*innen von Basisorganisationen und Aktivist*innen gefährdet sind. Dabei wird das Konzept der *lideres sociales* und Menschenrechtsverteidiger*innen in Kolumbien erläutert. Anschließend werden Gewaltdynamiken und politische Faktoren identifiziert, die vor und nach dem Friedensabkommen (2016) die Verteidigung von grundlegenden Menschenrechten zu einer lebensgefährlichen Aufgabe machen. Im zweiten Abschnitt werden die staatliche Schutzpolitik, Regelungen und Programme dargestellt, die vor und nach der Unterzeichnung des Friedensabkommens zum Schutz der Menschenrechtsaktivist*innen implementiert wurden. Der dritte Abschnitt analysiert die verschiedenen Faktoren auf operationeller, konzeptioneller und struktureller Ebene, die das kolumbianische Schutzparadigma kurz- und langfristig zu einer ineffektiven Politik machen. Im Fazit werden Schlussfolgerungen zu den Widersprüchen und Risiken der kolumbianischen staatlichen Schutzpolitik dargestellt.

## Der gewalttätige kolumbianische Frieden

Kolumbien ist seit mehr als drei Jahren damit befasst, ein Friedensabkommen umzusetzen, mit dem der über fünfzig Jahre andauernde interne bewaffnete Konflikt mit der größten Guerillagruppe des Landes, den Revolutionären Streitkräften Kolumbiens – FARC –, beendet werden sollte. Auch wenn offizielle Zahlen darauf hinweisen, dass die Zahl der bei Militäraktionen getöteten Zivilist*innen, an denen die FARC und die kolumbianischen Sicherheitskräfte beteiligt waren, in den ersten zwei Jahren nach der Unterzeichnung des Friedensabkommens zurückgegangen ist, hat die politische Gewalt gegen Zivilgesellschaft, Basisorganisationen und soziale Bewegungen rapide zugenommen (Human Rights Watch 2020).

**Abb. 1:**
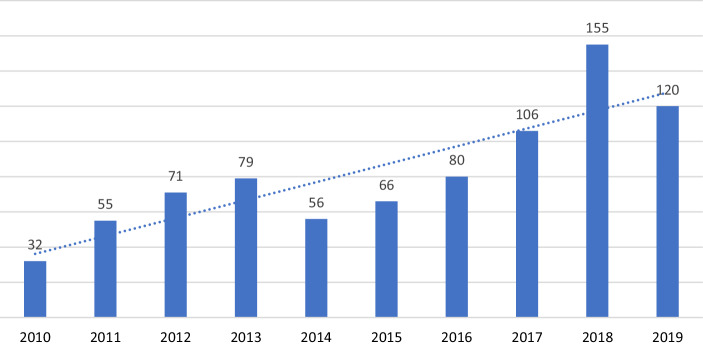
Ermordete Vertreter*innen von Basisorganisationen und Menschenrechtsaktivist*innen in Kolumbien zwischen 2010 und 2019. *Quellen*: Somos Defensores 2010, S. 4, 2017, S. 6, 2018, S. 9, 2019, S. 47; Nodal 2019

Die Anzahl der Angriffe und Morde unterschiedet sich je nach Quelle teilweise deutlich. Seit der Unterzeichnung des Friedensabkommens wurden zwischen 360 und 700 Aktivist*innen und Journalist*innen getötet (Indepaz 2019; siehe Abb. 1). Obwohl die genaue Anzahl der Angriffe und Morde nicht das Hauptthema dieses Artikels ist, fällt der große Unterschied zwischen den Zahlen der kolumbianischen Regierung, der Organisationen der Zivilgesellschaft und des Hochkommissars der Vereinten Nationen für Menschenrechte auf. Beispielweise wurden nach Regierungsangaben im Jahr 2019 107 Aktivist*innen ermordet (Consejeria Presidencial 2020, S. 10) während das Programm *Somos Defensores* die Zahl mit 120, INDEPAZ mit 234 (Nodal 2019) und das VN Hochkommissariat mit 108 (OACHNUDH 2020, S. 5) beziffern. Die Diskussion über die genaue Anzahl ist jedoch nicht entscheidend, da selbst die niedrigste, von der Regierung gemeldete Zahl alarmierend ist und ein systematisches Muster der Verfolgung dieser Gruppen beschreibt (Delgado 2017), das von der Regierung permanent geleugnet wird (Fiscalía General de la Nación 2016; El Espectador 2018). Die meisten Mordfälle sind dabei auf lokale Konflikte um Land und natürliche Ressourcen zurückzuführen (Dueholm Rasch 2017; Menig und Dietz 2020).

### Wer gilt als Menschenrechtsverteidiger*in?

Einer der Gründe für die Differenzen bei den oben genannten Zahlen ist die Diskussion darüber, wer in Kolumbien als Menschenrechtsverteidiger*in gilt. Dies ist zum Teil auf die weit gefasste Definition in der UN-Erklärung von 1998 zurückzuführen (Figari Layús 2020). Während sich in der UN-Erklärung der Begriff „Menschenrechtsverteidiger*in“ weder im offiziellen Titel noch im Erklärungstext selbst findet, definiert die Interamerikanische Menschenrechtskommission (IAKMR) das Konzept von Menschenrechtsverteidiger*innen wie folgt: „jede Person, die in irgendeiner Weise die Verwirklichung der auf nationaler oder internationaler Ebene anerkannten Menschenrechte und Grundfreiheiten fördert oder anstrebt“ (IAKMR 2006, S. 4).^1^ Diese weit gefasste Definition umfasst sowohl diejenigen, die sich beruflich für Menschenrechte einsetzen, als auch diejenigen, die informell und aus persönlichen, sozialen und politischen Gründen und auch nur gelegentlich mit der Verteidigung der Menschenrechte verbunden sind (IAKMR 2019, S. 21). Im Fall Kolumbiens wird der Begriff „líder/esa social“ synonym für „Menschenrechtsverteidiger*in“ verwendet. Dieses kolumbianische Konzept, das von den Vereinten Nationen als „Menschenrechtsverteidiger*in“ anerkannt wurde, bezieht sich auf verschiedene Arten der politischen, sozialen und kommunalen Führung. Dazu zählen z. B. Vertreter*innen von *Juntas de Acción Comunal* (JAC), Kleinbäuer*innen, afro-kolumbianische und Landrechts- und Umweltaktivist*innen, indigene Gemeinschaften, marginalisierte Gruppen, Journalist*innen, Aktivist*innen, die sich für die Rechte der aufgezählten Gruppen engagieren, sowie diejenigen, die sich für den Friedensprozess einsetzen, wie auch Teile der Zivilgesellschaft, die sich kritisch und unabhängig äußern (Guevara 2019; IAKMR 2019; Villamizar Acosta 2019).

Die Diskussion darüber, wer ein*e Menschenrechtsverteidiger*in ist, ist nicht nur auf die Unbestimmtheit des Begriffs in den verschiedenen internationalen Abkommen zu dem Thema zurückzuführen, sondern auch auf den politischen Gebrauch, den Regierungen von eben dieser Vagheit machen. Obwohl – wie oben aufgezeigt – ein breites Spektrum an Personen und Tätigkeiten als Menschenrechtsverteidiger*innen verstanden werden könnte, wird der Begriff von staatlichen Institutionen sehr oft restriktiv ausgelegt. Die mangelnde Anerkennung, aber auch das begrenzte Verständnis davon, was als Menschenrechtsarbeit bezeichnet werden kann, scheint bei bestimmten Gruppen von Aktivist*innen – wie z. B. Vertreter*innen von sozialen Bewegungen oder zivilgesellschaftlichen Organisationen bzw. Personen, die sich im Bereich Land und Territorium engagieren, und Personen, deren Lebensunterhalt nicht aus ihrer Arbeit als Menschenrechtsverteidiger*in herrührt – besonders ausgeprägt zu sein (Figari Layús 2020, S. 14–15). Mithilfe der Beschränkung der Definition wird der behördliche Ausschluss vieler Personen von Schutzmaßnahmen gerechtfertigt (Baeyens et al. 2015, S. 29), während gleichzeitig die Zahl der Angriffe gegen sie in der Öffentlichkeit kleingeredet wird, indem diese Angriffe als gewöhnliche und unpolitische Straftaten dargestellt werden.

### Grundlegende Faktoren der Gewalt gegen die Zivilgesellschaft in Kolumbien

Obwohl in den letzten Jahren die Zahl der Angriffe gegen die Zivilgesellschaft und kritische soziale Bewegungen zugenommen hat, sind diese Gewaltmuster in Kolumbien, aber auch in der gesamten Region Lateinamerika, nicht neu (Prem et al. 2018). Diejenigen, die heute als „Menschenrechtsverteidiger*innen“ und „soziale Vertreter*innen“ bezeichnet werden, unterscheiden sich nicht grundsätzlich von den früheren sozialen und politischen Akteur*innen, wie Gewerkschafter*innen, kritischen Oppositionellen, Journalist*innen, Intellektuellen und sozialen Organisationen, die in den 1960er, 1970er und 1980er Jahren unter den Doktrinen der nationalen Sicherheit und der Aufstandsbekämpfung auf dem gesamten Kontinent verfolgt wurden. Tatsächlich wurde in Kolumbien in den 1980er Jahren eine ganze politische Partei, die Patriotische Union (*Unión Patriótica*), durch die Ermordungen ihrer Mitglieder ausgelöscht. Die Verfolgung von gesellschaftlichen und kommunalen Vertreter*innen nahm insbesondere Ende der 1980er und Anfang der 1990er Jahre in Kolumbien mit der Entstehung der paramilitärischen Gruppen zu, die als Instrument gegen die „Subversion“ angesehen wurden (Gallón et al. 2013). Soziale Aktivist*innen wurden selektiv ermordet, während große Teile der Zivilbevölkerung gleichzeitig kollektiv vertrieben wurden. Dieser Modus Operandi galt als ergänzende Strategie, die insbesondere von Paramilitärs zur Gebietskontrolle eingesetzt wurde (Steele und Schubiger 2018). Jahre später, insbesondere unter der Regierung Álvaro Uribes (2002–2010), konzentrierte sich die Gewalt gegen die Zivilgesellschaft insbesondere, aber nicht nur, auf Gewerkschaftsmitglieder, die damals gegen das Freihandelsabkommen mit den USA protestierten (Prem et al. 2018). So zeigen sich „Gewaltzyklen“ gegen kritische zivilgesellschaftliche Akteure je nach Zeitraum und politischen Ereignissen mit mehr oder weniger Sichtbarkeit und Virulenz. Historisch und im Laufe der verschiedenen Regierungen haben sich die Bezeichnungen, die Methoden der Verfolgung oder die thematischen Angriffsprojekte verändert, aber die Logik der Gewaltausübung gegen Sektoren, die alternative Projekte vorschlagen oder die Macht der Eliten infrage stellen, bleibt konstant.

Der kolumbianische Fall zeigt ein auffälliges Merkmal mehrerer Post-Friedensabkommen^2^‑Szenarien: Auf die Beendigung bewaffneter Auseinandersetzungen folgen häufig fortgesetzte oder neue Formen von Gewalt. *Peace building* bei einem internen bewaffneten Konflikt ist per se ein langwieriger und komplexer Prozess. Diese Schwierigkeiten werden verschärft, wenn der Konflikt von mehreren Netzwerken und Konfliktparteien getragen wird, diese aber nicht alle am Verhandlungstisch einbezogen werden (Licklider 2001; Franke und Öztüurk 2015; Konig et al. 2017). Dies ist auch in Kolumbien der Fall. Während der direkte Konflikt zwischen der kolumbianischen Regierung und der FARC infolge des Abkommens endete, wurden weitere bewaffnete Gruppen wie die Nationale Befreiungsarmee (*Ejército Nacional de Liberación-ELN*), kriminelle Banden ehemaliger paramilitärischer Gruppen, die Volksbefreiungsarmee (*Ejército Popular de Liberación – EPL*) und Dissident*innen der FARC, die sich gegen ein Abkommen mit der Regierung aussprachen, von den Verhandlungen ausgeschlossen. Folglich wird der kolumbianische Friedensprozess oft als „ein partieller Friedensprozess“ beschrieben (Prem et al. 2018, S. 1). Unter solchen Umständen gibt es keine Garantie für ein Ende der politischen Gewalt, welche sich vorrangig auf die ländlichen Gebiete des Landes konzentriert, in denen die FARC einst die Kontrolle hatte. Damit verbunden sind mehrere Faktoren, die bei der Analyse der Zunahme der Gewalt gegen *lideres sociales* berücksichtigt werden müssen. Dazu zählen:

1. *Der Kampf um die Kontrolle über das Territorium:* Die von der FARC hinterlassenen Territorien wurden von einer geringen und ineffektiven staatlichen Präsenz oder ganz ohne diese übernommen. Aufgrund des Scheiterns, institutionelle Kapazitäten effektiv und rechtzeitig in diesen Gebieten aufzubauen, entstand dort ein Machtvakuum. In diesen Gebieten konkurrieren mehrere bewaffnete Gruppen (paramilitärische kriminelle Banden, Gruppen der organisierten Kriminalität, Dissident*innen der FARC sowie die ELN und die EPL) um die Kontrolle, Ausbeutung und den Gewinn aus illegalen wirtschaftlichen Aktivitäten (Prem et al. 2018). Die paramilitärische Gewalt sowie die Wahrnehmung anderer bewaffneter Akteur*innen, dass soziale Aktivist*innen ihre kriminellen Aktivitäten und wirtschaftliche Machtinteressen behindern, sind Faktoren, die eine wichtige Rolle bei der Zunahme der Gewalt spielen und den Friedensprozess gefährden können (Matallana 2018; Maher und Andrew 2018).
2. *Unzulängliche Wiedereingliederungspolitik*: Die geringe Wirksamkeit der Reintegration von ehemaligen und nun demobilisierten Guerillakämpfer*innen hat oft zur Rückkehr in den Krieg und zur Beteiligung an gewalttätigen Aktivitäten beigetragen. Nach Angaben der New York Times sind bis Mitte 2019 mindestens 3000 ehemalige Kämpfer*innen in den bewaffneten Kampf zurückgekehrt (Casey 2019). Unter dem Slogan der „unerfüllten Versprechen“ kritisierten demobilisierte Mitglieder der FARC die Wiedereingliederungspolitik, die durch den fehlenden Zugang zu öffentlichen und finanziellen Dienstleistungen, Wohnraum, Nahrung und wirtschaftlichen Möglichkeiten in Verbindung mit schlechten sanitären Bedingungen gekennzeichnet ist. Das Fehlen konkreter Pläne für den Wiedereingliederungsprozess kann die Beteiligung der FARC am Friedensprozess insgesamt erschweren. Und auch wenn dies nicht der Hauptfaktor für die Zunahme der Gewalt ist, hat die Problematik der mangelhaften Wiedereingliederung doch in mehreren Fällen zur Desertion und zur Wiederaufnahme illegaler Aktivitäten geführt (Schrimp 2018, S. 8).
3. *Soziale Ungleichheiten und die Prekarität der staatlichen Institutionen: *Nach Angaben der Wirtschaftskommission für Lateinamerika und die Karibik (*CEPAL*) ist Kolumbien eines der drei Länder mit der höchsten sozialen Ungleichheit in Lateinamerika (CEPAL 2018, S. 21). Gleichzeitig gehören die Gebiete, in denen die meisten Angriffe auf Menschenrechtsverteidiger*innen stattfinden, zu den am stärksten marginalisierten des Landes. Von der Gesamtzahl der Morde an sozialen Vertreter*innen geschahen im Jahr 2019 75 % in ländlichen Gebieten und 86 % in Gebieten mit einer Armutsrate, die über dem nationalen Durchschnitt liegt. Diese Gebiete sind in 98 % der Fälle auch durch die Anwesenheit illegaler Wirtschaftszweige und illegaler bewaffneter Gruppen gekennzeichnet (OACHNUDH 2020, S. 5). Obwohl es sich nicht um eine Bedingung handelt, die in allen Kontexten gleichbedeutend ist, ist in diesem Fall der Zusammenhang von Armut, Marginalität, Gewalt und der Abwesenheit des Staates als Bedingung für den fehlenden Zugang zu grundlegenden Rechten und Bedürfnissen nicht zu leugnen. Die fehlende Überwindung von Armut und sozialen Ungleichheiten, der eingeschränkte Zugang zu wirtschaftlichen Ressourcen, die Marginalisierung großer Teile der Bevölkerung und der Mangel an Möglichkeiten in diesen meist ländlichen Gebieten zeigen, dass die Prekarität der staatlichen Präsenz einer der wichtigsten Faktoren für die Entwicklung illegaler wirtschaftlicher Aktivitäten dort ist – und damit auch für die Zunahme der Gewalt.

## Die Widersprüchlichkeit der Schutz- und Sicherheitspolitik der kolumbianischen Regierung

Während der beiden Regierungsperioden von Santos (2010–2018) gab es bedeutende Fortschritte bei der Suche nach Schutzmaßnahmen für Vertreter*innen sozialer Organisationen durch die Unterstützung bestehender und neuer Schutzstrategien und Dialogräume zwischen der Regierung und der Zivilgesellschaft. In diesem Zusammenhang sind der „Runde Tisch für Sicherheitsgarantien“ (*Mesa Nacional de Garantías*) und die Gründung der „Nationalen Schutzeinheit“ (*Unidad Nacional de Protección – UNP*) im Jahr 2011 zu nennen. Der Runde Tisch für Sicherheitsgarantien findet im Rahmen des Nationalen Garantieprozesses für die Arbeit von Menschenrechtsverteidiger*innen und Sozial- und Gemeindevertreter*innen (*Proceso Nacional de Garantías para la labor de las defensoras y los defensores de derechos humanos, líderes sociales y comunales*) statt, der im April 2009 als Vereinbarung zwischen staatlichen Institutionen und nationalen sowie internationalen zivilgesellschaftlichen Organisationen verabschiedet wurde. Ziel des Prozesses war es, die Analyse der Menschenrechtssituation und die Entwicklung und Durchführung von Maßnahmen zur Prävention von, zum Schutz vor und zur Sanktionierung bei Gewalt voranzutreiben. Darüber hinaus sieht das Friedensabkommen unter Punkt drei die Gewährleistung der Sicherheit und die Bekämpfung und den Abbau von kriminellen Organisationen, einschließlich aller paramilitärischen Gruppen und ihrer Unterstützungsnetzwerke, vor. Dafür legt das Abkommen die Verabschiedung und Implementierung von Sicherheitsmaßnahmen fest. Um dieses Ziel zu erreichen, wurden in den Jahren 2017 und 2018 verschiedene Organe und Initiativen gegründet. Dazu gehören die „Nationale Kommission für Sicherheitsgarantien“ (*Comisión Nacional de Garantías de Seguridad*)^3^ und die „Sonderermittlungseinheit des Generalstaatsanwalts“ (*Unidad Especial de Investigación de la Fiscalía General de la Nación*).^4^

Obwohl dieser rechtliche Rahmen noch keine konkrete Lösung für das Problem der Gewalt an sozialen Aktivist*innen darstellt, zielt er auf die Ermöglichung eines offenen Dialogs zwischen der Regierung und der Zivilgesellschaft, um die bestehenden Schutzmechanismen zu verstärken und die Bereitstellung von Instrumenten zur Ermittlung und Prävention zu diskutieren und zu implementieren (Guevara 2019, S. 10). Dieser Dialogprozess wurde jedoch durch den Regierungswechsel 2018 und die Machtübernahme Iván Duques von der rechtskonservativen Partei Demokratisches Zentrum (*Centro Democrático*) unterbrochen (Gamboa Gutierrez 2019). Der neue Präsident ist für seine extrem kritische Haltung gegenüber dem Friedensabkommen und seine Nähe zum ehemaligen Präsidenten Álvaro Uribe bekannt. Duques Regierung darf zwar das Friedensabkommen nicht aufkündigen, schwächt jedoch systematisch dessen Umsetzung. Der im November 2018 vorgestellte „Nationale Entwicklungsplan“ (*Plan Nacional de Desarollo 2018–2022, PND*) zeigt deutlich die Prioritäten der Regierung. Das Friedensabkommen scheint nicht dazuzugehören. Deutliches Zeichen hierfür ist die Kürzung von Finanzmitteln für den Friedensprozess: die Sondergerichtsbarkeit für den Frieden (*Jurisdicción Especial para la Paz – JEP*), die Wahrheitskommission (*Comisión para el Esclarecimiento de la Verdad – CEV*) und die Einheit zur Suche von verschwundenen Personen (*Unidad de Búsqueda de Personas desaparecidas – UPBD*) haben eine Budgetkürzung von jeweils 28 %, 40 % beziehungsweise 68 % des beantragten Budgets erfahren (Valdez Correa 2019).

Das Sicherheitsprogramm der Regierung wird von einer neoliberalen wirtschaftlichen Agenda ergänzt, die auf Ressourcenabbau und -export basiert. Internationalen Konzernen soll die Ausbeutung der natürlichen Ressourcen (Bodenschätze und Agrargüter) erleichtert werden. Für die Umsetzung des Friedensabkommens im ländlichen Raum stellt die Regierung hingegen kaum Mittel zur Verfügung. In diesem Rahmen hat Duque im Februar 2019 einen sehr vagen „Plan für die Sicherheit und Verteidigung“ (*Plan de Defensa y Seguridad, PDS) *seiner Regierung präsentiert. Laut dem Präsidenten handelt es sich um einen umfassenden, mehrdimensionalen Ansatz, der die Sicherheitsprobleme des Landes bekämpfen soll. Menschenrechtsorganisationen kritisieren die Sicherheitspolitik hingegen als Neuauflage der „demokratischen Sicherheit“ (*seguridad democrática*) unter dem ehemaligen Präsidenten Uribe (Vásquez 2010). Die neue Sicherheitspolitik soll, wie schon früher in Kolumbien, zivile Informant*innen zur Bekämpfung illegaler Gruppierungen anwerben und für ihre Informationen belohnen.

Mit Fokus auf den PAO und die UNP werden im Folgenden zwei wichtige Dilemmata analysiert, welche die aktuelle kolumbianische Schutzpolitik ausmachen und die Ambivalenz der staatlichen Handlungen in Kolumbien zeigen. Die Schutzpolitik zeichnet sich durch drei Aspekte aus: 1. eine harte und unflexible Perspektive, die auf Militarisierung und der Verwendung eines polizeilichen, physischen Sicherheitsansatzes basiert, 2. die Exklusion der Zivilgesellschaft und der Opfer der Gewalt bei Entscheidungsprozessen – sowohl in der Planung als auch bei der Umsetzung der Maßnahmen – und 3. die begrenzte Wirksamkeit der Schutzmaßnahmen.

### Der Plan zur angemessenen Aktion: Militarisierung als Schutz?

- *Ein militarisiertes Schutzkonzept*

Generell reagiert die Regierung von Präsident Duque auf zivilgesellschaftliche Sicherheitsprobleme vor allem mit Militarisierung. Auf Makroebene sollte der seit November 2018 vom Innenministerium umgesetzte Aktionsplan zum Schutz von Menschenrechtsverteidiger*innen, sozialen und kommunalen Vertreter*innen und Journalist*innen (*Plan de Acción Oportuna de Prevención y Protección para los Defensores de Derechos Humanos, Líderes Sociales, Comunales y Periodistas, PAO*) zur Beendigung der Gewalt gegen soziale Aktivist*innen sowie zur Verbesserung der Präventions- und Schutzmaßnahmen beitragen (Ministerio del Interior 2018). Der Plan beinhaltet 18 Instrumente und gesetzliche Rahmenbedingungen zur Vorbeugung von Menschenrechtsverletzungen, von denen viele bereits seit mehreren Jahren existieren. Das allgemeine, von der Regierung proklamierte Ziel ist die Stärkung der Garantien für die Ausübung der Vertretung von Basisorganisationen und die Verteidigung der Menschenrechte. Theoretisch würde dies bedeuten, staatliche Institutionalität in jene Territorien zu bringen, die, wie oben erwähnt, wenig oder gar keine staatliche Präsenz haben. Eine weitere Achse des Plans ist die Entwicklung einer Kommunikationsstrategie gegen die Stigmatisierung von sozialen Führungskräften. In der Praxis sieht der PAO aber als einen seiner Schwerpunkte die Militarisierung und Stärkung der Streitkräfte in den Regionen Kolumbiens vor, in welchen die meisten sozialen Aktivist*innen angegriffen werden.^5^ Der PAO zielt auf die Lösung der Gewaltsituation bei gleichzeitiger größerer militärischer Kontrolle des Territoriums ab. Die Militarisierung sollte sich auf bestimmte, von der Regierung als *Strategische Zonen* (*Zonas Estratégicas de Intervención Integral – ZEII*) betrachtete Regionen konzentrieren. Die Regierung wählte hierfür acht Gebiete des Landes aus und argumentierte, es handele sich um Gebiete mit hoher Gewalt und einer starken Präsenz des Drogenanbaus: Cauca, Norte de Santander, Putumayo, Valle del Cauca, Huila, Antioquia, Bolivar und Choco.

Dieser militarisierte Ansatz ignoriert, dass Gewalt gegen Menschenrechtsverteidiger*innen ein strukturelles Problem und eine Folge der Komplexität des bewaffneten Konflikts ist. Die Perspektive des PAO ignoriert die Verantwortung staatlicher Beteiligter, indem sie sich nur auf private Akteure konzentriert. Diese Perspektive basiert auf der Annahme, dass paramilitärische Gruppen völlig unabhängige Akteure seien, die keine Verbindungen zum Staat hätten (Somos Defensores 2019). Der PAO leugnet so das von Unterstützung, Komplizenschaft und sogar gemeinsamen Aktionen geprägte Verhältnis, das die paramilitärischen Gruppen und das Militär seit Beginn des Paramilitarismus gepflegt haben (CINEP 2004; García-Peña 2004). Daraus resultiert die starke Ablehnung dieser militarisierten Schutzpolitik bei den zivilgesellschaftlichen Akteuren. Folgendes erklärt ein Mitglied einer Menschenrechtsorganisation in Bogotá, die mit Organisationen in anderen Regionen des Landes zusammenarbeitet:

Der PAO ist der größte menschenrechtliche Rückschlag, den wir erlebt haben. (…) Beim PAO handelt es sich im Grunde um militarisierte Maßnahmen, die sich angeblich auf die Gebiete konzentrieren, in denen die meisten Angriffe gegen Menschenrechtsverteidiger*innen stattgefunden haben. (…) Aber wir haben diese stärkere Militarisierung der Territorien abgelehnt (…) Der PAO wird all diejenigen, die von den Paramilitärs bedroht werden, die überall in Koordination, Koexistenz und Komplizenschaft mit dem Militär handeln, niemals schützen. Die Aktivist*innen werden ermordet, weil sie beispielsweise (…) Anspruch auf vom Paramilitarismus geraubtes Land erheben, oder weil sie die Straflosigkeit von Militärkommandanten oder Politikern, die mit dem Paramilitarismus in Verbindung stehen, anprangern. (Interview 1 durchgeführt im März 2019, Bogotá)

Staatliche Sicherheitsansätze, die für den Kampf gegen kriminelle Netzwerke auf Militarisierung setzen, schaffen ebenfalls ein gefährliches Umfeld für Menschenrechtsverteidiger*innen. Wie kann jemand das Militär als Akteur des (persönlichen) Schutzes wahrnehmen, wenn es für viele der Angriffe, die die Menschenrechtsverteidiger*innen erleben, mitverantwortlich ist oder den Paramilitarismus ungestraft agieren lässt? Insbesondere in den ländlichen Gebieten sind die Beziehungen zwischen dem Militär und den paramilitärischen Kräften bis heute sehr stark ausgeprägt (Grajales 2017; Ronderos 2014).

b. *Exklusion der Zivilgesellschaft von Entscheidungsprozessen*

Die mangelnde Flexibilität und Berücksichtigung der Perspektiven und Bedürfnisse der zu schützenden Menschen zeigt sich auch auf nationaler Ebene bei der Gestaltung und Umsetzung der Sicherheitspolitik. Mehrere Befragte erwähnten einen allmählichen Prozess der Ausgrenzung der Zivilgesellschaft unter der Regierung von Iván Duque. Die aktuelle Regierung ignoriere den partizipatorischeren Ansatz des ehemaligen Präsidenten Santos, der die Zivilgesellschaft seit dem Friedensabkommen in die Gestaltung der Politik miteinbezogen hatte:

Mit Santos gab es einen Dialog und Respekt. Es war nicht gerade effizient, aber es herrschte Einigkeit. (…) Die Frage des Schutzes von Menschenrechtsverteidiger*innen hatte zwei sehr wichtige Instanzen, den Runden Tisch für Sicherheitsgarantien und die Nationale Kommission für Sicherheitsgarantien, die unter Beteiligung der Regierung, der Delegierten der Zivilgesellschaft und der internationalen Gemeinschaft stattfanden. Jetzt funktionieren sie (diese Instanzen) nicht mehr. Die aktuelle Regierung hat uns (nur) einmal eingeladen, und es war eine Verspottung (…). (Interview 4 durchgeführt im März 2019, Bogotá)

Dialogräume wie der „Runde Tisch für Sicherheitsgarantien“ oder die „Nationale Kommission für Sicherheitsgarantien“ wurden praktisch gestrichen, und die neuen Regelungen und Programme, die theoretisch dem Schutz sozialer Aktivist*innen dienen sollen, schlossen die Zivilgesellschaft von ihrer Gestaltung aus. Tatsächlich besteht der durch den PAO geschaffene Ausschuss nur aus Regierungsbeamt*innen und -institutionen wie dem Präsidenten, den Innen- und Verteidigungsministerien, der Armee, der Polizei und der Schutzeinheit UNP (Ministerio del Interior 2018, S. 3). Im Gegensatz zu früheren Dialogräumen gibt es keine Stimmen von Vertreter*innen zivilgesellschaftlicher Organisationen. Die Zusammensetzung des Ausschusses zeigt den ausschließlich militärischen und polizeilichen Charakter von Duques Schutzpolitik. So beschreibt der PAO einen Wechsel zu einer militarisierten und unilateralen, nationalen Schutzpolitik der Exklusion. Während die Regierung von Juan Manuel Santos die „Kommission für Sicherheitsgarantien für den Abbau des Paramilitarismus“ gegründet hat, wie im Friedensabkommen vereinbart, zielt die derzeitige Politik des PAO auf die Militarisierung der ländlichen Regionen ab und schließt die Zivilgesellschaft als grundlegende Akteurin der Entscheidungsprozesse über Schutzpolitiken strukturell aus.

### Die Nationale Schutzeinheit des Innenministeriums: Mit dem Feind zusammenleben?

- *Polizeiliche Natur des Schutzes*

Ähnlich wie der PAO zielt auch die Nationale Schutzeinheit UNP auf individuellen Schutz. Die UNP wurde im Jahr 2011 gegründet, um Schutz- und Präventionsmaßnahmen für Menschenrechtsverteidiger*innen und andere gefährdete Gruppen bereitzustellen. Diese Art von Programmen ist in Kolumbien nicht neu, was auch die historische Natur und Persistenz des Problems zeigt. Kolumbien war sogar das erste Land in Lateinamerika, das 1997 ein nationales Programm zum Schutz von Menschenrechtsverteidiger*innen eingerichtet hat. Dieses ist in modifizierter Form bis heute in Kraft. Das „Programm zum Schutz von Menschenrechtsverteidigern, Gewerkschaftern, Journalisten und sozialen Aktivisten“ konzentriert sich auf den Schutz des Rechts auf Leben, Freiheit und persönliche Sicherheit gefährdeter Gruppen (Congreso de Colombia 1997; Dekret 418, Art. 49).

Das kolumbianische Schutzprogramm ist durch eine starke Bürokratisierung gekennzeichnet, die für viele unmittelbar bedrohte Menschen die Beantragung und Bearbeitung des Schutzes zu einem langen und risikoreichen Prozess macht. Die Bearbeitung eines Schutzantrags kann bis zu 90 Tage dauern, bis dieser dem „Ausschuss für Risikobewertung und Handlungsempfehlungen“ (*Comité de Evaluación de Riesgo y Recomendación de Medidas – CERREM*) vorliegt. Nach einer Risikoanalyse entscheidet dieser Ausschuss, welche Maßnahmen der bedrohten Person gewährt werden. Bis zur Umsetzung können bis zu 60 Tage vergehen (IAKMR 2017, S. 99–111).

Wie die kolumbianische Sicherheitspolitik auf nationaler Ebene ist das Schutzmodell der UNP auf einen „polizeilichen Ansatz“ beschränkt (Carvalho et al. 2016, S. 179; Amnesty International 2017, S. 6). Die Maßnahmen umfassen, je nach Fall, die Begleitung durch bewaffnetes Sicherheitspersonal, ein gepanzertes Auto, Panikknöpfe, Überwachungskameras, Satelliten-Handys, schusssichere Westen sowie finanzielle Unterstützung für den Fall, dass die Person aus Sicherheitsgründen in eine andere Stadt ziehen muss (Quintana und Eguren Fernandez 2011, S. 107). Die Bereitstellung solcher Maßnahmen für jede betroffene Person wird als „Sicherheitsschema“ (*Esquema de Seguridad*) bezeichnet. Im August 2020 stellte die UNP 7350 Menschen Sicherheitsschemen zur Verfügung. Von diesen 7350 Individuen sind 4978 *lideres sociales, *das sind 68 % all derjenigen, die Sicherheitsmaßnahmen erhalten (Presidencia de la República 2020). Die Mehrheit dieser Maßnahmen wird individuell gewährt. Andere Hilfen wie medizinische, soziale und psychologische Unterstützung wurden von diesem Programm bisher nicht in Erwägung gezogen. Das zum Schutz eingesetzte Personal stammt – entgegen der Empfehlungen des IAKMR (2017, S. 161) – in der Regel von privaten Sicherheitsfirmen und hat in vielen Fällen einen polizeilichen oder militärischen Hintergrund.

Durch die Akzeptanz der Teilnahme am Schutzprogramm der UNP und die Zuweisung von Personenschutz entsteht ein tägliches Zusammenleben, das dem Sicherheitspersonal Zugang zur gesamten Arbeitsagenda sowie zur privaten, sozialen und familiären Sphäre der zu beschützenden Person ermöglicht. Obwohl nicht ausgeschlossen ist, dass ein Vertrauensverhältnis zwischen beiden Personen aufgebaut wird, sind die meisten dieser Beziehungen durch Misstrauen und Verdächtigungen gekennzeichnet, die konkrete Veränderungen im Leben der geschützten Personen bedeuten. Die meisten der Befragten, die Sicherheitsmaßnahmen erhalten haben, sind in der Tat der Ansicht, dass das eingesetzte Sicherheitspersonal ihnen nicht nur Schutz bietet, sondern auch Spionageaufgaben wahrnimmt und die Sicherheitskräfte über ihre Aktivitäten informiert, was für sie wiederum ein Risikofaktor ist. Tatsächlich hat es mehrere Skandale in Kolumbien gegeben, die die Mehrfachfunktion des Personenschutzes deutlich gemacht haben: Schutz, Überwachung und Spionage (Duque 2018). Die Angst davor, von ihrem Personenschutz ausspioniert zu werden, führt zu Veränderungen und Einschränkungen im täglichen Leben der geschützten Personen. Mehrere Befragte sagten, dass sie den Personenschutz nicht über alle ihre Aktivitäten informieren, insbesondere wenn sie mit ihrer politischen oder Menschenrechtsarbeit zu tun haben. Beispielweise berichtet ein Menschenrechtsverteidiger aus Bogotá, der außergerichtliche Hinrichtungen des Militärs bearbeitet, von den Vorsichtsmaßnahmen, die er auf sich nimmt, wenn er mit seinem Personenschutz täglich zur Arbeit fährt:

*(mit den Schutzmaßnahmen) ist das Leben sehr eingeschränkt. (…) Ich weiß, dass ich in meiner Kommunikation sehr vorsichtig sein muss. Wenn ich im Auto (mit dem Personenschutz) bin, kann ich nicht über alles reden. Vor allem, wenn ich von Beweisen weiß, von Fällen, in welche hohe Armeekommandeure involviert sind … Dann sind das Dinge, über die man nicht reden kann. (…) Obwohl ich dem Personenschutz bis zu einem gewissen Grad vertrauen kann, kann ich nicht wissen, ob er Berichte weitergibt*. (Interview 1 durchgeführt im März 2019, Bogotá)

Das Misstrauen gegenüber dem eingesetzten Sicherheitspersonal wird noch dadurch verstärkt, dass viele Begünstigte des Schutzprogramms zuvor von staatlichen Sicherheitskräften bedroht oder attackiert worden sind. Außerdem gibt es Vorwürfe gegen Personal des Personenschutzes, in verschiedene Verbrechen wie z. B. sexualisierte Gewalt verwickelt zu sein: So erklärt eine Journalistin aus Medellín, die viele Zweifel hinsichtlich der Schutzmaßnahmen der UNP hat:

*Ich war sehr ehrlich zur UNP und sagte: „Hören Sie, ich traue der Polizei nicht, denn die Polizei in Medellín ist verdorben. Ich meine, ich werde dies tun (die Schutzmaßnahmen akzeptieren), aber ich traue dem Staat nicht und ich traue auch der UNP nicht. Ein UNP-Personenschützer vergewaltigte hier in Medellín eine Journalistin, und es gibt viele UNP-Personenschützer, die Informanten sind.” *(Interview 2 durchgeführt im März 2019, Medellín)

Verschiedene Frauen haben Beschwerden über Belästigungen und sexuelle Übergriffe durch den Personenschutz eingelegt (Duque 2018). Es scheint jedoch schwierig zu sein, weibliches Begleitpersonal zu gewinnen. Als alleinerziehende Mutter mit einer Tochter bat die oben zitierte Journalistin wiederholt um einen Wechsel der Begleitpersonen und betonte, dass sie weibliche Schutzpersonen wünsche. Dieser Antrag wurde stets abgelehnt.

*Ich sagte (in der UNP), dass ich mich unwohl fühle, weil sie (die Wachen) zwei Männer sind und ich bin eine Frau und obendrein eine alleinerziehende Mutter mit einer Tochter. Und ich habe ständig mit meinem kleinen Mädchen zu tun, und manchmal muss man Unterwäsche kaufen … Und ich begann, das irgendwie nicht mehr zu machen. Oder als ich in ein Einkaufszentrum ging, um eine Jacke anzuprobieren, musste meine Tochter draußen (mit den Wachen) bleiben. Und ich habe kein Vertrauen, sie mit zwei Männern allein zu lassen. Ich sagte deswegen, dass (…) ich eine Frau als Schutzperson brauche. Aber die Leute der UNP sagten mir, nein, dass (…) es keine Änderung geben kann. *(Interview 2 durchgeführt im März 2019, Medellín)

Den Schutzmaßnahmen mangelt es an einer intersektionalen und geschlechterbasierten Perspektive. Wenn es sich bei einer Person beispielsweise zugleich um eine Frau, alleinerziehende Mutter und eine Journalistin und Aktivistin handelt, ist sie aufgrund ihres Berufes, ihres Geschlechts und ihrer familiären und sozialen Situation mehrfach gefährdet, und benötigt differenzierte Maßnahmen. Dies ist besonders schwerwiegend in einem Land mit ständigen Angriffen sowohl auf die Presse, als auch auf weibliche Aktivistinnen. Laut der Stiftung für Pressefreiheit (*Fundación para la Libertad de Prensa* – FLIP) haben die Angriffe gegen und Einschüchterungen von Journalist*innen seit der Unterzeichnung des Friedensabkommens in Kolumbien rasant zugenommen (FLIP 2020, S. 3). In den letzten drei Jahren (2017 bis 2019) wurden 583 Journalist*innen in Kolumbien bedroht, während in der vorangegangenen Dreijahresperiode (2014–2016) 257 bedroht wurden (FLIP 2020, S. 3).

Seit 2011 umfasst das kolumbianische Schutzprogramm formell und rechtlich einen „differenzierten Ansatz“ (Ministerio del Interior 2011), der sowohl bei Risikobewertungen als auch bei der Implementierung von Schutzmaßnahmen unter Berücksichtigung von Aspekten wie Alter, Ethnizität, Geschlecht, Behinderung, sexueller Orientierung und Herkunft angewandt werden sollte. Wie die Befragten jedoch mitteilten (und wie von verschiedenen Organisationen berichtet wurde), wird dieser differenzierte Ansatz in der Praxis nicht angewandt (IAKMR 2017, S. 107, 108).

b. *Exklusion der Gewaltopfer von Entscheidungsprozessen*

Eine Maßnahme für mehr Vertrauen und mehr Sicherheit könnte in einer größeren Beteiligung der von Gewalt Betroffenen bei Entscheidungsprozessen bezüglich ihrer spezifischen Schutzmaßnahmen bestehen, z. B. einer stärkeren Beteiligung an der Auswahl des Sicherheitspersonals. Obwohl einige Befragte sagten, sie dürften die Lebensläufe der möglichen Kandidat*innen im Voraus sehen, war dies in den meisten Fällen nicht möglich. Die Mehrheit der Maßnahmen wird allgemein und unflexibel formuliert und berücksichtigt kaum die Bedürfnisse, Lebensumstände und Aktivitäten der betroffenen Person, Organisation oder Gruppe. Die „geschützten Personen“ werden als Objekte, nicht als aktive Subjekte wahrgenommen. Sie werden in ein sogenanntes Schutzschema (*esquema de protección*) gesteckt, in dem es sehr schwierig ist, sich zu bewegen, und in dem die Perspektive des Gewaltopfers nicht berücksichtigt wird. Mehrere Befragte beschrieben, dass die ihnen zugewiesenen Schutzmaßnahmen nicht an ihre Familie, ihr Geschlecht, ihre Arbeit oder ihren Glauben angepasst wurden. Ein Menschenrechtsverteidiger aus Bogotá erzählte, dass die gewährten Schutzmaßnahmen ihm zwar ein gewisses Gefühl der Sicherheit vermittelten, gleichzeitig aber sein tägliches Leben und das seiner bei ihm lebenden Tochter veränderten und einschränkten:

*F: Waren die Schutzmaßnahmen für Sie und Ihre Familie?*

*A: Ja, für mich und meine Tochter, mit der ich zusammenlebte. Es war sehr schwierig, weil meine Aktivitäten sich von denen meiner Tochter unterschieden. Wir hatten nur eine Wachperson und nur ein Auto. Und es war sehr kompliziert, etwa wenn sie in der Universität war und ich um 8 Uhr morgens im Büro sein musste. (…) Es ist eine anstrengende Situation. Das gilt auch für die Situation, dass man nachts nicht ausgehen kann. Da Sie wissen, dass Sie früh zu Hause sein müssen, schließen Sie sich früh ein. Und für mich war es nicht so schwer, (…), aber meine Tochter, die bereits 19 Jahre alt war … Sie zu bitten, sich abends um 18 oder 19 Uhr einzusperren, ist unmöglich, nicht wahr? Von einer jungen Frau zu verlangen, nicht mit ihren Freunden auf die Straße zu gehen, ist schwierig. *(Interview 1 durchgeführt im März 2019, Bogotá)

Während es die Situation der Bedrohung ist, die die Lebensqualität und Möglichkeiten der Aktivist*innen sowohl in ihrem beruflichen als auch in ihrem privaten Bereich stark einschränkt und modifiziert, trägt die Starrheit des Schutzprogramms wenig zu einer Besserung bei. Die mangelnde Flexibilität bezüglich der Lebensbedingungen und Präferenzen der geschützten Personen scheint eine Konstante bei der Umsetzung des Programms zu sein, die sich noch verschärft, wenn die zu schützende Person eine Frau ist. Die bereits erwähnte Journalistin und Aktivistin aus Medellín, die zwei Männer als Wachpersonal zugeteilt bekommen hatte, sagte, die zwei Männer seien unflexibel, hätten sie angelogen, seien oft betrunken und hätten sie sogar misshandelt, was ihr mehr Anlass zur Sorge als Schutz gebracht habe. Genauso wie die Maßnahmen nicht den individuellen Bedürfnissen der zu schützenden Person im Sinne von Familiensituation, Geschlecht oder Tätigkeit entsprechen, verhält es sich ähnlich im Fall der politischen und religiösen Einstellungen oder bezüglich des persönlichen Sicherheitsgefühls. Dies berichtete beispielsweise ein Menschenrechtsverteidiger, der mit seiner Organisation in ländlichen Gebieten arbeitet. Nachdem er Opfer wiederholter Drohungen und Verfolgungen wurde, baten er und seine Organisation die UNP darum, dass die ihm zugeordneten Wachmänner nicht bewaffnet sein sollten. Obwohl diese Bitte zunächst respektiert wurde, änderte die UNP nach einer Weile die Bedingung. Sie schickten ihm und der Organisation bewaffnetes Sicherheitspersonal, was bei ihm ein größeres Gefühl der Unsicherheit hervorrief. Seiner Überzeugung nach erhöht das Tragen einer Waffe das Risiko von Gewalt.

*Wir haben die UNP um unbewaffneten Personenschutz gebeten. (…), weil es unsere Überzeugung ist, dass die Anwendung von Gewalt nicht zu unserem Schutz eingesetzt werden sollte (…). Aber was passierte dann? Dass die zunächst unbewaffneten Schutzmaßnahmen plötzlich zu bewaffneten Schutzmaßnahmen wurden, ohne mich zuvor zu konsultieren. Dann erschien eines Tages der Wachmann bewaffnet (…). Als ich davon erfuhr, (…) schickte ich eine Mitteilung (…) an die Nationale Schutzeinheit mit unseren Argumenten (…). Die Antwort war, dass der Schutz nur dann garantiert sei, wenn das Schutzpersonal bewaffnet ist (…). (*Interview 3 durchgeführt im März 2019, Bogotá)

Ein vertrauensvolles Verhältnis zwischen „Beschützenden“ und „Geschützten“ ist ein wichtiger Faktor für den Programmerfolg. Vor allem wenn man bedenkt, dass die zu schützenden Menschen bereits oftmals Opfer mehrfacher Gewalt waren, die in vielen Fällen ihre Lebensplanung verändert und sogar unterbrochen hat. Damit die Schutzmaßnahmen wirksam sind und ein Gefühl von Schutz und Sicherheit mit sich bringen, ist es daher wichtig, dass sich zwischen dem Personenschutz und der zu schützenden Person ein Vertrauensverhältnis entwickelt. Dies kann nur auf der Grundlage eines partizipativen und ganzheitlichen Prozesses erreicht werden, der die Bedürfnisse, Werte und Meinungen der bedrohten Person oder der zu schützenden Organisation sowie den politischen und territorialen Kontext, in dem sich die Person befindet, einbezieht. Ein solcher „relationaler Ansatz“ (Eguren Fernández und Patel 2015, S. 902) bedeutet die Berücksichtigung des jeweiligen Kontexts und der komplexen Realitäten des Lebens und des Alltags der Menschenrechtsverteidiger*innen und Journalist*innen in Kolumbien.

Die erschwerte Partizipation von Verteidiger*innen zeigt sich nicht nur in Einzelfällen, sondern auch in der Zusammensetzung der Entscheidungsgremien des Schutzprogramms, wie z. B. dem CERREM, dem Ausschuss, der Risiken bewertet und die Schutzmaßnahmen beschließt. Das Problem der standardisierten Maßnahmen und nicht partizipativen Entscheidungsprozesse der UNP wurde bereits von der Interamerikanischen Menschenrechtskommission thematisiert. Sie hob die mangelnde Flexibilität bei der Umsetzung der Maßnahmen hervor; die Notwendigkeit einer stärkeren Beteiligung und Konsultation der Opfer bei der Festlegung der Schutzsysteme sowie die Notwendigkeit, die Kommunikation und Zusammenarbeit zwischen den verschiedenen für den Schutz zuständigen Stellen zu verbessern (IAKMR 2017, S. 110). Doch auch Jahre nach dieser Empfehlung bleibt das Schutzmodell nicht nur unverändert, sondern wird auch in der nationalen Sicherheits- und Schutzpolitik repliziert. Dieses Vorgehen trägt nicht zur Vertrauensbildung und zum Dialog zwischen den Parteien bei.

Zudem geht das Misstrauensverhältnis über das Sicherheitspersonal oder die Schutzpolitik hinaus. Es bezieht sich auch auf eine historische und konfliktreiche Beziehung zwischen dem kolumbianischen Staat und kritischen zivilgesellschaftlichen Akteur*innen, die weitgehend auf der Konfrontation von Interessen und unterschiedlichen Weltanschauungen bezüglich Entwicklungsmodellen und dem Zugang zu Menschenrechten beruht. Die kolumbianische Politik hat oft gezeigt, dass kritische soziale Akteure nicht als wichtige Beteiligte an sozialem Wandel, Entwicklung und Demokratie, sondern eher als Feinde des Staates beziehungsweise der dominanten Gruppen innerhalb des Staates wahrgenommen werden. Dies führt bis heute zu einer Politik der Verfolgung, Stigmatisierung und Kriminalisierung. In diesem Prozess der Marginalisierung und Ausgrenzung – der Zeiten eines engeren Dialogs wie unter der Regierung Santos erlebt hat – war die Rolle der Sicherheitskräfte zusammen mit den paramilitärischen Gruppen, mit denen es historische Beziehungen der Unterstützung beziehungsweise Toleranz gab, von entscheidender Bedeutung. Die systematische Straffreiheit der Angriffe ist auch ein Zeichen der Toleranz gegenüber diesen Verbrechen und damit der Verantwortung des Staates. In diesem Rahmen ist es verständlich, dass die soziale Dynamik der Schutzpolitik auf Makro- und Mikroebene durch Misstrauen gekennzeichnet ist, da dieses nicht einfach von der historischen Rolle des kolumbianischen Staates dekontextualisiert und abgekoppelt werden kann.

## Die Unwirksamkeit der Schutzpolitik

Die aktuelle Schutzpolitik der kolumbianischen Regierung steht vor mehreren Herausforderungen. Generell ist die Schutzpolitik sowohl im Falle des PAO als auch im Falle der UNP durch einen hohen Grad an Ineffektivität, Misstrauen und oft mangelnder Legitimation durch ihre Zielgruppe, die Menschenrechstaktivist*innen, gekennzeichnet. Als Schlussfolgerung lassen sich diese Schwierigkeiten in der folgenden Tab. 1 zusammenfassen, die eine Pyramide mit drei Ebenen darstellt. Die erste, operative Ebene ist für Faktoren und strukturelle Probleme in Kolumbien verantwortlich, die von der Schutzpolitik ausgeschlossen werden und die für die Beendigung der Ursachen von Angriffen auf Aktivist*innen entscheidend sind. Die zweite Zeile der Tabelle bezieht sich auf die konzeptionellen Faktoren, die eine effektive, legitime und integrale Schutzpolitik bereits in der Planungsphase behindern. Die dritte Zeile erklärt die wichtigsten operationellen Herausforderungen, mit welchen sich die konkrete Umsetzung bestehender Schutzmaßnahmen konfrontiert sieht.

- *Die strukturelle Ebene*

**Tab. 1:** Mehrschichtige Mängel des Schutzparadigmas. *Quelle*: eigene Darstellung

Ebene	Defizite/Schwierigkeiten
Operativ	– Mangel an finanziellen Mitteln – Verzögerungen bei der Anwendung von Sicherheitsmaßnahmen – Unwirksamkeit verschiedener Schutzmaßnahmen
Konzeptionell	– Standardisierter Ansatz zum Schutz verschiedener Gruppen – Begrenzte Beteiligung der Zivilgesellschaft
Strukturell	– Keine Durchführung von Ermittlungen – Keine Bekämpfung der Ursachen von Gewalt

Zwar ist die Behebung von Mängeln in der Planungs- und Umsetzungsphase der Schutzpolitik der Schlüssel zur Verbesserung der Sicherheit von Menschenrechtsverteidiger*innen, doch sind solche Maßnahmen noch immer kurzfristig und gehen nicht auf die ursächlichen Faktoren politischer Gewalt ein. In diesem Sinne ist die Kontinuität der politischen Gewalt gegen soziale Aktivist*innen sowie ehemalige FARC-Kombattant*innen der wichtigste Indikator für die Ineffektivität dieser Schutzpolitik. In der Tat ist der Paramilitarismus derzeit, eineinhalb Jahre nach Verabschiedung des PAO, gestärkt. Die von der FARC verlassenen Gebiete wurden von illegal bewaffneten und kriminellen Gruppen besetzt. Das staatliche Vorgehen sowie wirtschaftliche und soziale Alternativen scheinen bisher nicht ausreichend oder gänzlich ineffektiv zu sein. Fast vier Jahre nach dem Friedensabkommen hat es keine Reform der Land- oder Ressourcenverteilung gegeben und die sozialen Ungleichheiten sind nach wie vor enorm – eine Situation, die sich durch die Eindämmungsmaßnahmen aufgrund der Corona-Pandemie zusätzlich verschärft hat (EL Espectador 2020a).

Ein weiteres grundlegendes Problem der Sicherheitspolitik besteht in dem geringen Engagement dafür, Täter*innen zu ermitteln und zu sanktionieren. Diane Orentlicher (2005, S. 6) definiert Straflosigkeit als:

*die Unmöglichkeit, *de jure* oder *de facto*, Straftäter zur Rechenschaft zu ziehen, ob in Straf‑, Zivil- oder Disziplinarverfahren, da sie keiner Ermittlung unterworfen werden, die dazu führen könnte, dass sie angeklagt, verhaftet, und, falls sie schuldig gesprochen werden, zu angemessenen Strafen verurteilt werden und ihre Opfern Entschädigung zuteil wird*.

Die Straflosigkeit in Kolumbien ist sehr hoch und strukturell bedingt. Sie liegt im Kontext von Angriffen gegen Menschenrechtsaktivist*innen bei etwa 98 % (*Serna Duque* 2019; OACHNUDH 2019, S. 8, 9). Das Problem der Straflosigkeit ist nicht nur ein rechtliches, sondern auch ein politisches. Die Straflosigkeit kann *de jure* oder *de facto* sein. *De jure* ergibt sich Straflosigkeit direkt oder indirekt aus Gesetzen oder Vorschriften, wie z. B. Amnestien oder Begnadigungen. Im Gegensatz dazu liegt *de facto* Straflosigkeit vor, wenn ein bestehendes Gesetz aufgrund sozialer oder politischer Zwänge in der Praxis nicht angewandt und dadurch Strafverfolgung und Bestrafung behindert werden (Ambos 1999, S. 34). Im Falle von Angriffen auf soziale Vertreter*innen herrscht in Kolumbien *de facto* Straflosigkeit. Bürokratie, Korruption, Nachlässigkeit und mangelnder politischer Wille spielen dabei grundlegende Rollen. Alle Befragten, die bereits Opfer von Bedrohungen wurden, äußerten, dass sie keinerlei Antwort auf ihre eingereichten Beschwerden erhalten haben. In keinem der Fälle wurden Angreifer*innen bzw. Täter*innen verhaftet oder die Hintergründe der Angriffe ermittelt. In einigen Fällen führten die Opfer eigene Ermittlungen durch und reichten sogar Beweise bei der Staatsanwaltschaft ein. Doch auch Jahre später haben sie keine Antwort der Justiz erhalten. Dies war z. B. der Fall bei einem Befragten, der im Jahr 2014 in der Innenstadt von Bogotá von zwei Angreifern auf der Straße attackiert, bedroht und dessen Laptop mit wichtigen Informationen gestohlen wurde.

*F*^6^*: Gab es Fortschritte bei den Ermittlungen der Staatsanwaltschaft bezüglich des Überfalles?*

*A*^7^*: Nein. Und es kamen nicht nur dort wo, der Angriff stattgefunden hat, Leute vorbei, sondern es gab auch Kameras. Es gab 13 Kameras rund um den Ort des Angriffs (…). Ich übergab der Staatsanwaltschaft die Information über diese 13 Kameras und wo sie zu finden waren. Ich habe nach den Informationen über den Standort der Kameras gesucht, weil die Staatsanwaltschaft selbst es nicht machen wollte. Ich habe ihnen gesagt, wo die Kameras stehen. Und ich sagte ihnen: „Bitte gucken Sie sich die Videos an, es ist sehr einfach. (…) Schauen Sie nach den Kameras.“ (Aber) Sie haben nichts getan. (…) Zwei Jahre später sagte mir die Staatsanwaltschaft, dass sie die Kameras gesehen hätten und keine Videos mehr vom Tag des Angriffes dabei gewesen seien*. (Interview 1 durchgeführt im März 2019, Bogotá)

Schwierigkeiten wie diese und Ausreden aller Art, wie z. B. exzessive Langsamkeit bei den Ermittlungen, Mangel an Ressourcen oder Personal, übermäßige Bürokratie oder „verlorene“ Beweise sind Hindernisse, mit denen viele derjenigen, die Angriffe und Drohungen erlitten haben, ab dem Zeitpunkt ihrer Anzeige bei der Staatsanwaltschaft und der Polizei konfrontiert sind. Meistens bleiben solche Beschwerden ergebnislos.

Die Situation zeigt noch einmal, dass weder die Formulierung noch die Anwendung (oder auch Nichtanwendung) des Gesetzes objektiv sind. Sie werden in jedem Kontext von gesellschaftlichen und politischen Interessen und Bedürfnissen beeinflusst. Guillermo O’Donnell hat analysiert, dass sich in Lateinamerika die Anwendung des Rechts dadurch kennzeichnet, dass sie als wirksames Mittel der Unterdrückung kritischer und marginalisierter Bevölkerungsgruppen nach freiem Ermessen angewandt wird (O’Donnell 1998). Die Kehrseite davon ist die Art und Weise, in der sich die privilegierten Sektoren, sei es direkt oder durch entsprechende persönliche Verbindungen, von der Einhaltung des Gesetzes befreien. In diesem Zusammenhang wird die Gewalt des Staates, der Eliten oder mächtiger privater Akteure wie paramilitärischer Gruppen oder Wirtschaftsunternehmen in der Regel nicht dem regulären Strafrecht unterworfen. Diese selektive Anwendung des Gesetzes zeigt sich deutlich in dem Umgang mit Angriffen auf Menschenrechtsverteidiger*innen in Kolumbien. Während die Täter*innen solcher Verbrechen ein hohes Maß an Straffreiheit genießen, ist die Kriminalisierung der Aktivist*innen in Kolumbien übliche Praxis (Hernández et al. 2019). Antony Duff (2001) zufolge fungiert das Gesetz als ein Instrument der Kommunikation über Werte und Regeln, die festlegen, welche Verhaltensweisen in einer bestimmten Gesellschaft erlaubt sind und welche nicht. Laut Duff ist die Relevanz der Botschaft des Gesetzes durch die Sanktion oder Bestrafung gegeben. Hier stellt sich die Frage, welche Botschaft die strukturelle Straflosigkeit in den Fällen von Angriffen auf Menschenrechtsverteidiger*innen an die Gesellschaft sendet und welche Konsequenzen dies hat. Wenn die Straflosigkeit in diesen Fällen strukturell wird, sendet dies eine Botschaft der Vernachlässigung und Unterschätzung aus, die zur Normalisierung beiträgt und ein Bild erzeugt, „als ob nichts geschehen wäre“ (Figari Layús 2017, S. 82–83). Diese Situation hat schwerwiegende Auswirkungen auf das Leben der Menschenrechtsverteidiger*innen. Die Psychologin Elina Aguiar (1993) führt aus, dass die Straffreiheit der Täter*innen dazu führt, dass die Betroffenen der Gewalt zu einer „Risikopopulation“ werden. Straffreiheit aufgrund fehlender Untersuchungen und ausbleibender strafrechtlicher Verfolgungen mutmaßlicher Verantwortlicher geht mit einer realen und potenziell konkreten Bedrohung und Gewalt gegenüber den Opfern durch diese Täter*innen einher. Die Folgen für die Betroffenen können dramatisch sein. Neben psychischen Folgen wie Angst und Gefühlen der Isolation berichten sie auch von negativen Auswirkungen der Beschuldigungen und Stigmatisierung ihres Ansehens in ihrem persönlichen oder sozialen Umfeld. Die Situation der Hilflosigkeit beeinträchtigt und schränkt die Qualität des täglichen Lebens der Betroffenen in vielerlei Hinsicht ein. Wie ein Befragter erzählt, hat ihn die Risikosituation – trotz Schutzmaßnahmen – dazu veranlasst, sein Leben enorm einzuschränken, sodass er zum Beispiel sein eigenes Haus zu einer Art „Bunker“ umgestaltet hat, in dem er jahrelang fast wie eingesperrt gelebt hat:

*Es ist wirklich eine sehr beunruhigende Situation, aber man gewöhnt sich am Ende fast daran. Ich wohne in einem Haus mit Gittern bis zur Decke und elektrischen Zäunen und Alarmanlagen. Fast ein Bunker, oder? Es ist eine schwierige Situation. Auch die Tatsache, abends nicht ausgehen zu können. Da man weiß, dass man früh zu Hause sein muss, schließt man sich früh ein. (…) Es ist auch ein besorgniserregendes Umfeld, das das alltägliche Leben betrifft, weil ich immer daran denken muss, dass ich einen Teil meines Einkommens für Schutz, Sicherheit, Mobilität, wie zum Beispiel immer mit einem Taxi zu fahren, aufwenden muss. Ich kann nicht die öffentlichen Verkehrsmittel benutzen oder allein in die Parks gehen. *(Interview 1, durchgeführt im März 2019, Bogotá)

In ständiger Angst zu leben, ist nicht einfach. Deshalb wird es angesichts der wahrgenommenen fehlenden Optionen normalisiert: „*Man gewöhnt sich daran“.* In Kolumbien, wo die Täter*innen ungestraft und frei leben und im schlimmsten Fall sogar weiterhin in staatlichen Institutionen und Sicherheitsapparaten arbeiten, leben die Aktivist*innen in einer latenten Risikosituation. Sehr schwierig sind die Ermittlungen in den Fällen von Angriffen, an denen Sicherheitskräfte, insbesondere das Militär, beteiligt sind. Diese Fälle werden oft an die Militärjustiz übergeben. Ein empörter betroffener Aktivist aus Bogotá erklärt:

(…) Die Staatsanwaltschaft führt keine Ermittlung durch bei Angriffen gegen Menschenrechtsverteidiger*innen, bei denen Sicherheitskräfte involviert sind. Warum? Weil es hier ein paralleles Justizsystem gibt, das Militärstrafrechtssystem, in dem die Vorgesetzten der an den Verbrechen beteiligten Militärs selbst die Ermittlungen durchführen. Die Militärjustiz ist eine „Freispruchs-Maschine“. Dort werden alle freigesprochen! Dies ist ein System der Komplizenschaft. Das ist ein System der Straflosigkeit. (Interview 1, durchgeführt im März 2019, Bogotá)

Die Straflosigkeit ist nicht nur abhängig davon, wer der*die Täter*in ist, sondern auch davon, wo die Verbrechen stattfinden. Besonders in ländlichen Gebieten sind viele Aktivist*innen zusätzlich Angriffen und Drohungen illegaler, bewaffneter Gruppen ausgesetzt (Oxfam 2019, S. 27). Ländliche Gebiete sind in Kolumbien weiter von mangelndem Zugang zur Justiz geprägt. Die begrenzte Präsenz der Staatsanwaltschaft in diesen Regionen wird am Mangel an materiellen, technischen und personellen Ressourcen sowie angemessenen Sicherheitsmechanismen deutlich. Solange Staaten nicht ernsthaft gegen die Verantwortlichen von Angriffen ermitteln und diese sanktionieren, bieten Schutzmaßnahmen nur eine schwache und kurzfristige Lösung, ohne dabei die strukturelle Problematik zu adressieren. Die Straflosigkeit steht dem Ziel der Nichtwiederholung strukturell entgegen.

b) *Die konzeptionelle Ebene*

Die dargestellten konzeptionellen Mängel der Schutzpolitik beziehen sich sowohl auf die Planung als auch auf die Konzeption dieser Schutzmaßnahmen, die sich danach in ihrer Umsetzung widerspiegeln. Wie oben beschrieben, gibt es zwei Hauptprobleme, die in dieser Phase hervorstechen: der standardisierte Ansatz zum Schutz verschiedener Gruppen und die begrenzte Beteiligung der gefährdeten Personen beim Entscheidungsprozess der Schutzpolitik und -maßnahmen. Tatsächlich hängen beide Aspekte miteinander zusammen, da es nicht möglich ist, angemessene Maßnahmen mit einer geschlechtsspezifischen, intersektionalen, geografischen und kulturellen Perspektive zu konzipieren, wenn die Menschen, die bestimmte Eigenschaften aufweisen und die durch die Maßnahmen geschützt werden sollen, nicht in die Planungsphase mit einbezogen werden. Die Umsetzung von standardisierten Maßnahmen ist leichter durchführbar, aber oft sehr ineffektiv. Der bereits genannte „relationale Ansatz“ (Eguren Fernández und Patel 2015, S. 902), der den jeweiligen Kontext und die komplexen sozialen und politischen Realitäten der Menschenrechstverteidiger*innen in die Planung und Implementierung von Schutzpolitiken einbezieht, würde eine sinnvollere und integrativere Konzeption des Schutzes ermöglichen.

c) *Die operative Ebene*

Von hoher Relevanz sind die operativen Schwierigkeiten, welche die konkrete Umsetzung von Schutzmaßnahmen behindern. In der Implementierungsphase geht es vor allem um praktische Faktoren, die mit bürokratischen und finanziellen Aspekten sowie mit dem schlechten oder verspäteten Einsetzen der gewährten Maßnahmen zusammenhängen. Eines der Schlüsselprobleme ist die knappe oder unzureichende Finanzierung der Maßnahmen, die sich unmittelbar auf ihre Wirksamkeit und ihren Umfang auswirkt. Die Knappheit von Finanzmitteln wird an zwei grundlegenden Aspekten sichtbar: zum einen an der Anzahl der angenommenen Schutzfälle und zum anderen an den gewährten Maßnahmen selbst. Es werden wesentlich weniger Fälle als schutzbedürftig akzeptiert und damit viel weniger Schutz gewährt, als Beschwerden vorgetragen werden (Figari Layús 2020). Auf der anderen Seite hat die mangelnde Finanzierung Personalmangel sowie niedrige Löhne zur Folge. In einigen Fällen haben die knappen finanziellen Mittel beispielsweise sogar zu institutionellen Krisen der UNP und Personalstreiks geführt (Revista Semana 2014; Pacifista 2019). Das Paradoxe daran ist, dass die UNP von allen Schutzprogrammen in Lateinamerika das größte Budget hat. Das jährliche Budget der UNP beträgt eine Billion kolumbianische Pesos (ca. 280 Mio. Dollar). Die Verwaltung und Zuteilung der Mittel durch die UNP wurde jedoch wegen mangelnder Kontrolle über die Verwendung der Mittel und wegen Korruptionsvorwürfen kritisiert (Cuesta 2019).

Mehrere operative Defizite bestehen in den verschiedenen Phasen des sogenannten UNP-Schutzzyklus, welche die Umsetzung ab dem Zeitpunkt der Beschwerde bis zur Einsetzung konkreter Schutzmaßnahmen verzögern. Zunächst sind dies längere Verzögerungen bei der Bearbeitung der Anträge, der Risikobewertung und der Umsetzung der Sicherheitsmaßnahmen. Damit verzögert sich auch – wenn der Fall angenommen wird – die entsprechende Gewährung von Schutzmaßnahmen. Während der Bearbeitungszeit sind die bedrohten Personen weiterhin einer strukturellen Risikosituation ausgesetzt. Werden Schutzmaßnahmen gewährt, sind bei der Umsetzung und Funktionsweise gravierende Mängel festzustellen. Dies betrifft Verzögerungen bei dringenden Schutzmaßnahmen (IAKMR 2017, S. 109, 110, 119). Außerdem entstehen Verzögerungen häufig bei der Zuweisung von Sicherheitspersonal sowie bei der Bereitstellung von Sicherheitskameras oder dem sogenannten Panikknopf. Das Fehlen oder die schlechte Funktionsweise der Notvorrichtungen können gravierende Auswirkungen auf die körperliche und psychische* Unversehrtheit *von gefährdeten Menschenrechtsverteidiger*innen haben, die damit in eine noch riskantere Situation gebracht werden. In einigen Fällen wurden Menschenrechtsverteidiger*innen ohne Personenschutz, aber auch in Begleitung von Schutzpersonal, ermordet (Isaza Giraldo 2017; Miranda 2019).

In diesem Zusammenhang stellt sich noch die Frage, warum die bedrohten Personen die Maßnahmen des Schutzprogramms fordern und akzeptieren. Haben diese noch eine andere Funktion, die zum Schutz beiträgt? Auf diese Frage antwortete ein Gewerkschafter aus Bogotá, der dem Schutzprogramm ebenfalls sehr kritisch gegenübersteht, es aber in Anspruch nimmt, Folgendes:

*Ich werde Ihnen sagen, was mir diese Sicherheitsmaßnahmen geben, was sie ermöglichen. Sie können zeigen, dass das Risiko wirklich besteht und dass der Staat es anerkennt. Und diese minimale Geste des Staates erlaubt es mir, meine Verteidigungsmechanismen sowie meine Anklagen zu stärken. (…) Diesen Schutz zu akzeptieren, war nicht einmal meine Idee. Aber auf diese Weise ist es belegt, dass ich in Gefahr bin, und das ist es, was ich wollte. *(Interview 5 durchgeführt im März 2019, Bogotá)

Der Erhalt des staatlichen Schutzes scheint auf diese Weise eine symbolische Funktion zu haben: Die staatliche Anerkennung ihrer Menschenrechtsarbeit als Grund für die Gewalt und ernste Risikosituation, mit der sie konfrontiert sind. Diese symbolische Anerkennung ist für viele der bedrohten sozialen Aktivist*innen von großer Bedeutung, insbesondere in Kontexten, in denen die Regierung von Iván Duque auch heute noch den systematischen Charakter von Angriffen auf Menschenrechtsverteidiger*innen leugnet und wo die Verteidigung von Menschenrechten häufig Gegenstand von Diskreditierung und Stigmatisierungskampagnen ist (Hernandez et al. 2019, S. 77–78).

## Fazit

Dieser Beitrag hat die Hauptmerkmale und Herausforderungen der umstrittenen kolumbianischen Sicherheitspolitik zum Schutz von gefährdeten Gruppen betrachtet. Der Schwerpunkt lag auf den *líderes sociales*. Hierbei hat sich dieser Artikel auf die folgenden Fragen konzentriert: Welche Rolle und Auswirkungen haben staatliche Schutzmaßnahmen in Kolumbien bei der Bekämpfung und Beendigung von Gewalt? Welche Eigenschaften und Herausforderungen weist diese Schutzpolitik auf? Welche Konsequenzen haben die Schutzmaßnahmen tatsächlich für ihre Zielgruppe? Die Analyse der Effektivität und Komplexität der Schutzpolitik fokussierte auf die Perspektive ihre Zielgruppe: Vertreter*innen sozialer Organisationen, Aktivist*innen und Journalist*innen.

Der kolumbianische Fall zeigt, dass eine staatliche Schutzpolitik für Menschenrechtsverteidiger*innen, die auf Militarisierung, Standardisierung und entkontextualisierten Maßnahmen sowie Ausschluss der Betroffenen aus dem Entscheidungsprozess basiert, nicht dazu beiträgt, Gewalt gegen und Stigmatisierung von sozialen Aktivist*innen zu reduzieren. In der Tat schafft diese Schutzpolitik bei vielen Menschen weder ein sicheres Umfeld für das Verteidigen von Menschenrechten, noch die Grundlagen zur Ausübung ihrer Arbeit. Im Gegenteil: Es schränkt das tägliche Leben der Aktivist*innen ein.

Auf längere Sicht würde eine ernsthafte Sicherheits- und Schutzpolitik die Bekämpfung der strukturellen Faktoren beinhalten, die der politischen Gewalt zugrunde liegen, bzw. eine ganzheitliche Perspektive des Schutzes verfolgen, die eine Politik der Umverteilung von Ressourcen wie Land und des Zugangs zu grundlegenden Dienstleistungen für marginalisierte Bevölkerungsgruppen gewährleistet. Ansätze zur „menschlichen Sicherheit“ (Krause 2005), die Unsicherheit als ein mehrdimensionales und strukturelles Phänomen begreifen, werden von der gegenwärtigen kolumbianischen Regierung nicht aufgenommen. In diesem Kontext führen Menschenrechtsverteidiger*innen weiterhin einen doppelten Kampf. Einerseits arbeiten sie an der Verteidigung ihrer Rechte und der Interessen ihrer Gemeinschaften. Andererseits sind sie mit einem ständigen und einsamen Kampf konfrontiert, um Bedrohungen auszuweichen und zu überleben. Obwohl die Symbolik von staatlichem Schutz wichtig ist, reicht diese nicht aus.

Das aktuelle kolumbianische Konzept des „Schutzes“ lässt im Sinne der menschlichen Sicherheit wichtige Aspekte von grundlegender Bedeutung unbeachtet. Der Schutz und der Sicherheitsansatz der kolumbianischen Regierung basieren auf der Komponente der physischen und militarisierten Sicherheit. Aber das Konzept von Schutz sollte eine breitere Perspektive einbeziehen, wenn man eine grundlegende und langfristige Strategie gegen die Gewalt gegen Aktivist*innen und Vertreter*innen sozialer Organisationen anstrebt. Eine integrale Schutzpolitik sollte sich auf die Sicherheit der Bevölkerung auf allen Ebenen ausrichten, das heißt, in Bereichen der Gesundheitsversorgung, Arbeit, Bildung, Lebensmittelversorgung, Verwaltung, Justiz, Umwelt, Sicherheit der Einzelnen und der Gemeinschaft. Die Konzipierung von „staatlicher Präsenz“ sollte nicht auf die Militarisierung des Landes reduziert werden, sondern von Prinzipien wie der „menschlichen Sicherheit“ geleitet sein.

Die Gewalt belastet den kolumbianischen Friedensprozess mit einer schweren Hypothek. Schließlich ist eine der Grundvoraussetzungen für den Übergang von einem bewaffneten Konflikt zu einer friedlichen Demokratie die Garantie der Nichtwiederholung. Der Aufbau des Friedens beruht nicht nur auf der Wiederherstellung des Gewaltmonopols durch den Staat, sondern auch auf der Inklusion marginalisierter Bevölkerungsgruppen und der Stärkung demokratischer, ziviler und staatlicher Institutionen. Ein Mittel, um diesen Prozess zu ermöglichen, ist die Öffnung und Anerkennung institutioneller und integrativer demokratischer Räume, um die uneingeschränkte Beteiligung und Meinungsäußerung der Gesellschaft und die Erfüllung der Menschenrechte zu fördern, die während des bewaffneten Konflikts systematisch verletzt wurden.

Die zunehmende Gewalt macht leider auch vor der Coronavirus-Pandemie nicht halt, wie die große Zahl an Tötungen von Mitgliedern sozialer Organisationen und politischer Bewegungen im Jahr 2020 zeigt (El Espectador 2020b). Zwischen Januar und September 2020 wurden 135 *lideres sociales* ermordet (El Espectador 2020c). Gerade weil sich die Risikosituation der Aktivist*innen im Kontext der Pandemie verschlechtert hat, sollten die Covid-19-Maßnahmen kein Vorwand sein, um Schutzmaßnahmen aufzuheben und bedrohte Personen weiterer Gefahr auszusetzen, wie viele Menschenrechtsorganisationen jüngst berichtet haben (Amnesty International 2020). Es gibt offenbar keine Quarantäne für Mörder*innen.

## Footnotes

1 Dieses Zitat wurde, wie die anderen Zitate im Text, von der Autorin übersetzt.

2 In diesem Text wird das Konzept des „Post-Konflikts“ nicht verwendet, da das Friedensabkommen nur mit der FARC Guerilla geschlossen wurde und andere bewaffnete Akteure, die noch aktive Parteien des Konflikts sind, unberücksichtigt lässt: z. B. andere Guerilla Gruppen wie die Nationale Befreiungsarmee (*Ejército Nacional de Liberación – ELN*) und die Volksbefreiungsarmee (*Ejército Popular de Liberación – EPL*) oder paramilitärische Gruppen.

3 Die durch das Dekret 154 geschaffene Kommission bestehend aus Regierung und zivilgesellschaftlichen Organisationen hatte ein ehrgeiziges Ziel: die Formulierung und Überwachung einer öffentlichen und strafrechtlichen Politik zum Abbau paramilitärischer Organisationen und krimineller Gruppen, die für Angriffe gegen Verteidiger*innen verantwortlich sind.

4 Andere vorgeschlagene Instanzen zur Stärkung der Sicherheit im Friedensabkommen sind: eine Eliteeinheit der Nationalen Polizei (*Cuerpo Élite en la Policía Nacional*), ein integrales Sicherheitssystem für die Ausübung der Politik (*Sistema Integral de Seguridad para el Ejercicio de la Política*), das umfassende Sicherheits- und Schutzprogramm für Gemeinschaften und Organisationen in den Regionen (*Programa Integral de Seguridad y Protección para las Comunidades y Organizaciones en los Territorios*) und die Maßnahmen zur Prävention und Bekämpfung der Korruption (*Medidas de Prevención y Lucha contra la Corrupción*).

5 Mit diesem Ziel rechnet der PAO mit der Beteiligung mehrerer Institutionen. Dazu gehören die Nationale Schutzeinheit (UNP), die Sektorübergreifende Kommission für eine schnelle Reaktion auf Frühwarnungen (*Comisión Intersectorial Para la Respuesta Rápida a Las Alertas Tempranas, CIPRAT*) und die Strategie zur Ermittlung und Verurteilung von Morden an Menschenrechtsverteidiger*innen und Mitgliedern sozialer Organisationen durch die Generalstaatsanwaltschaft.

6 Das „F“ bezieht sich auf die Frage der Interviewerin.

7 Das „A“ bezieht sich auf die Antwort des/der Interviewers/in.
